# Supplementary material for: Towards integration of time-resolved confocal microscopy of a 3D in vitro microfluidic platform with a hybrid multiscale model of tumor angiogenesis
Source: PLoS Comput Biol. 2023 Jan 18;19(1):e1009499. doi: 10.1371/journal.pcbi.1009499 (PMC9886306; doi:10.1371/journal.pcbi.1009499)
Supplement: S1 Appendix — In this appendix, we show the invertibility of the models by calibrating with in silico data. We then explore the accuracy of the Gaussian fits of the parameter distributions calibrated in Scenarios 1-5. The longitudinal prediction of the ABM is presented after calibration during Scenarios 1-4 and another region of local vasculature is presented. (DOCX) [file pcbi.1009499.s001.docx]

**S1. Appendix**

**Model Invertibility**

To ensure the invertibility of the multistep calibration method (i.e., that the calibrated models have a unique combination of parameters that give the best fit), we calibrate the systems with synthetically generated data. First, we do this in the ordinary system of equations for VEGF concentration and then in the agent-based model with longitudinal vascular density measurements. We generate data *via* Eqs. (1)-(4) at 24, 48, 72, 96, 120, 144, and 168 hours and then add 5% Gaussian noise to the data. We then utilize Bayesian calibration to calibrate the VEGF production rate, consumption rate, and carry capacity PDFs, shown in S1 Fig. The error in the maximum likelihood values is 9.3%, 2.0%, and 5.8% respectively, while the average error in the prediction is 3.8% and 5.9% for the tumor and endothelial cell experiments, respectively. For the ABM model, we generated synthetic data using the maximum likelihood values of VEGF production and consumption, calibrated in Scenario 1-2, and stalk cell divide time, calibrated in Scenario 3. We then calibrated the distance between new tip cells using the synthetic data (shown in S2 Fig). The calibration, in the presence of no additional noise, selected the exact parameter value used to generate the data, and the error between the maximum likelihood value and the mean of the fit Gaussian distribution was 9.8%.

**Gaussian fit of parameter distribution functions**

We assess the accuracy of using Gaussian fits to describe the calibrated parameter distribution functions. We characterize this accuracy *via* S1 Table, by showing the quantiles at 0.05, 0.25, 0.5, 0.75, and 0.95. These are calculated by interpolating the cumulative distribution function of the parameter distributions to the quantiles (i.e., the quantile value is where the cumulative distribution function is equal to the quantile). The quantiles are calculated using an interpolation scheme developed at (<https://github.com/wwarriner/matlab_quantiles>).

**Time evolved ABM solution**

In Scenarios 1-4, we inform essential model parameters from multimodal data through parameter calibration. We predict the angiogenic sprout length and the vascular density, both with uncertainty. In S3 Fig, we show the temporally evolved solution of one sample from the parameter distribution functions calibrated in Scenario 4 (the one-parameter case).

**Scenario 5: Local sprout elongation rate calibration region 2**

In S4 Fig and S5 Fig, we show the results of the calibration and prediction, respectively, of local region 2. In S4 Fig, Panels (A) - (C) show the best fit (red), the centerlines of the data (green), and the overlap (yellow), with the corresponding hybrid ABM prediction of days 3, 5, and 7 shown in Panels (D) - (F), respectively. Panel (G) shows the calibrated PDF (black) and the Gaussian fit of the local stalk cell growth rate (blue). The maximum likelihood value is 72.1 hours and the Gaussian fit is $d_{SC}$~ N(78.86,10.83) hours. In S5 Fig, we take 1000 samples from the Gaussian fit of stalk cell divide time and calculate the 1%, 5%, and 10% prediction envelopes of the model, shown in Panels (A)-(C), (D)-(F), and (G)-(I), respectively. The prediction quartiles of both local regions are shown in Table 5.
