# Supplementary figures and images for: Towards integration of time-resolved confocal microscopy of a 3D in vitro microfluidic platform with a hybrid multiscale model of tumor angiogenesis
Source: PLoS Comput Biol. 2023 Jan 18;19(1):e1009499. doi: 10.1371/journal.pcbi.1009499 (PMC9886306; doi:10.1371/journal.pcbi.1009499)

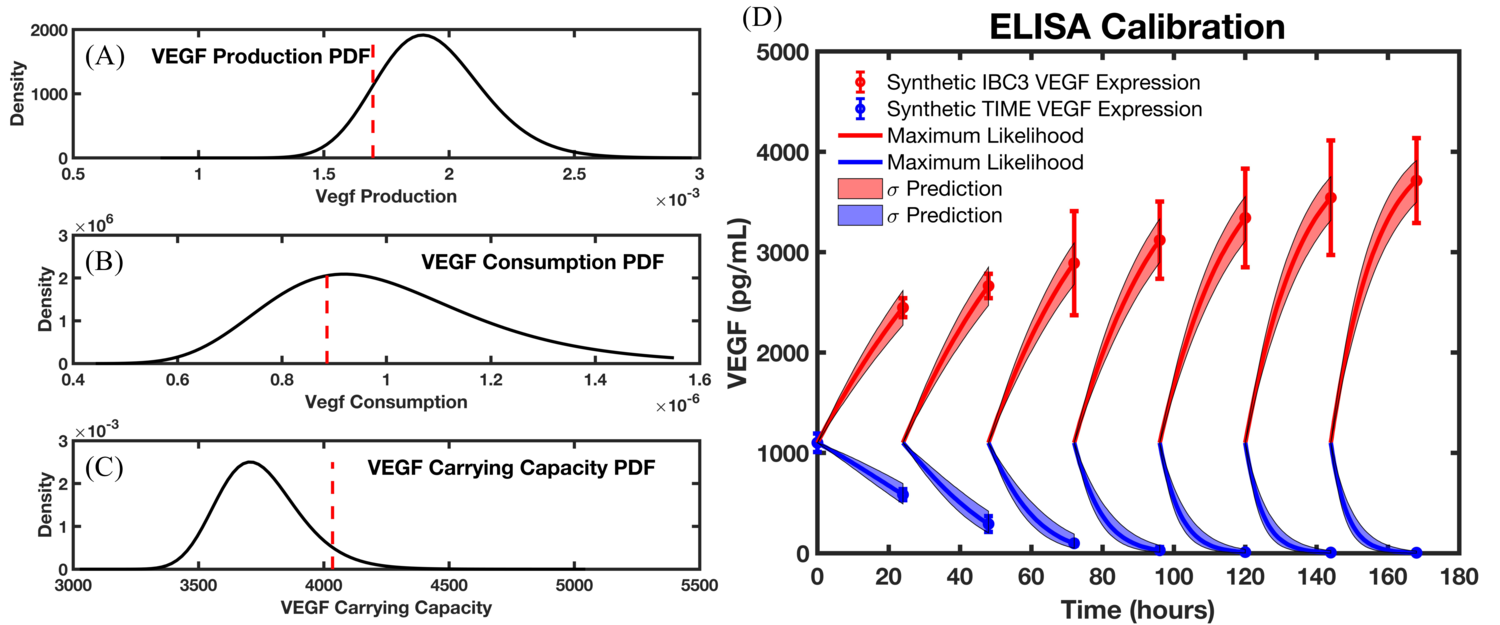

Supplement: S1 Fig — Panels (A)-(C) depict the parameter distributions from calibrating VEGF production, consumption, and carry capacity, respectively, to 5% Gaussian noised VEGF concentration data. Panel (D) shows the goodness of fit of the VEGF concentration in the tumor (IBC3) and endothelial (TIME) experiments, shown in red and blue, respectively. The relative error between the parameters used to generate the data and the calibrated parameters are 9.3%, 2.0%, and 5.8%. (TIF) [file pcbi.1009499.s002.tif]

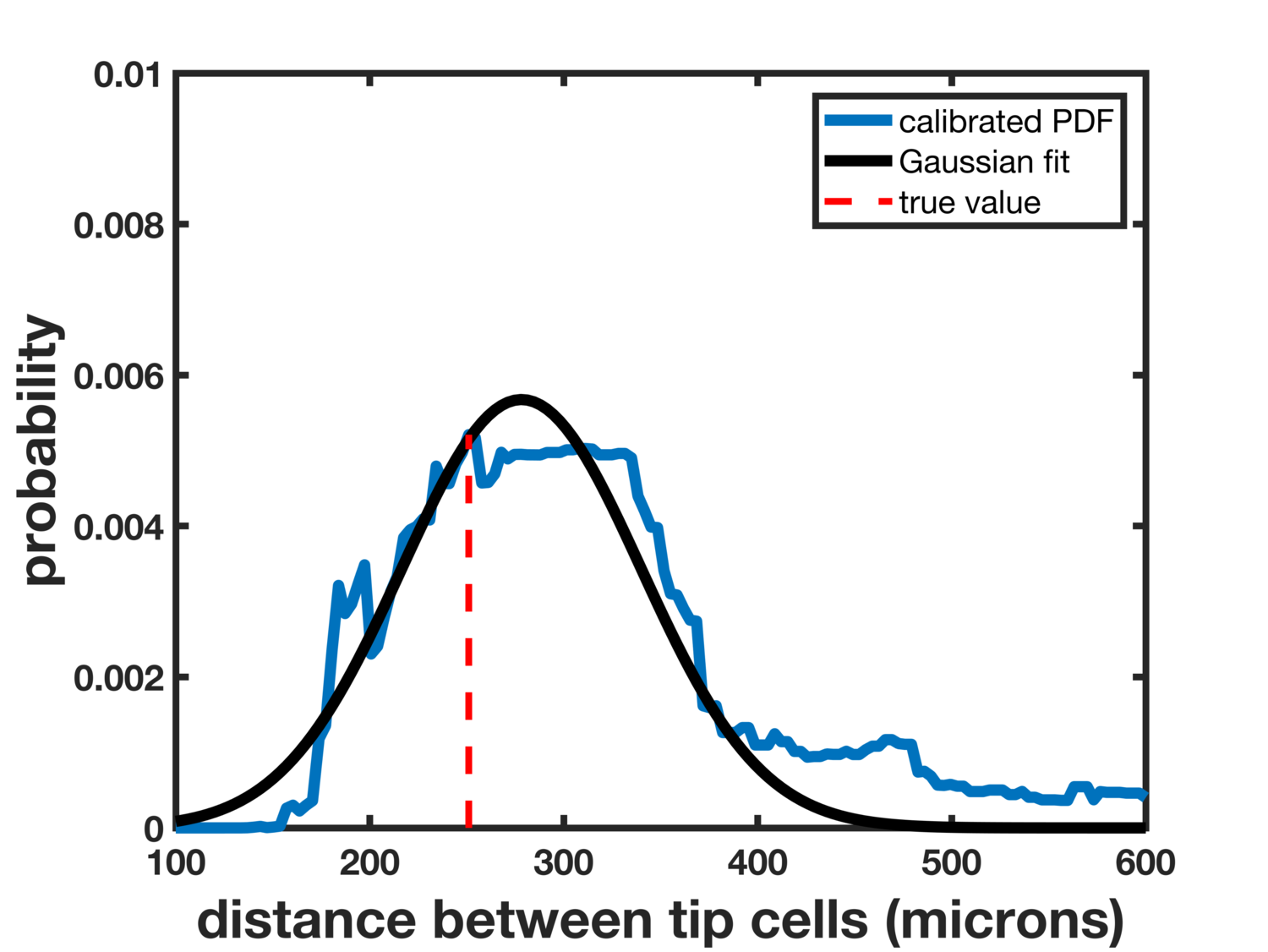

Supplement: S2 Fig — This plot shows the calibrated PDF when the model is calibrated against synthetic data. (TIF) [file pcbi.1009499.s003.tif]

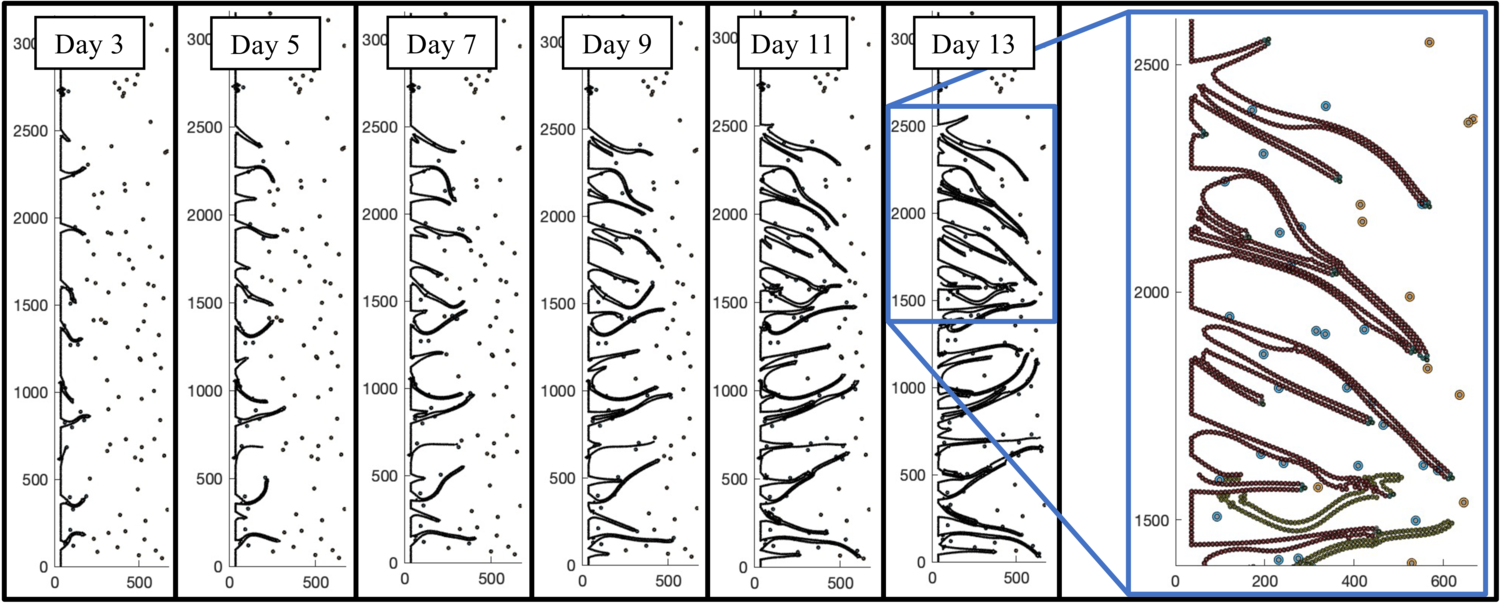

Supplement: S3 Fig — Endothelial cells—red, tip cells—green, deactivated endothelial cells—yellow green, normoxic tumor cells—blue, hypoxic tumor cells—orange. Days 3-13 are predicted using informed parameters calibrated in Scenarios 3 and 4. The x- and y-axis are in microns. The vasculature grows and develops in response to vascular endothelial growth factor, resulting in the final vascular network, with a highlighted region shown in blue. This region has several anastomosing vessels. (TIF) [file pcbi.1009499.s004.tif]

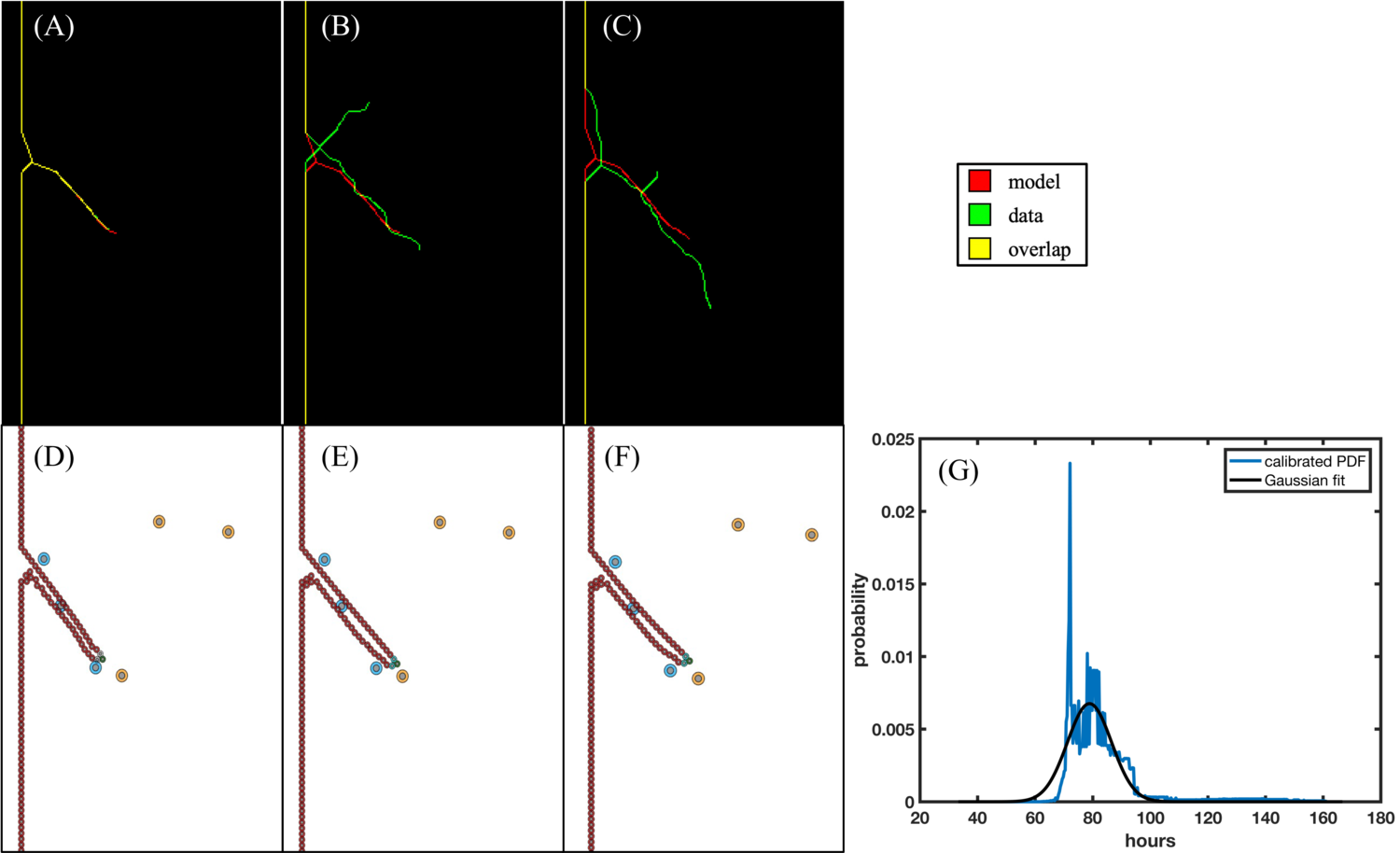

Supplement: S4 Fig — Panels (A)—(C) show the centerlines calculated from the model best fit in red, the data in green, and the overlap in yellow of day 3 (used to inform the initial conditions), Day 5, and day 7 (both used to calibrate the stalk cell growth rate). The agent-based model is shown in Panels (D)-(F) with tip cells in green, stalk cells in cyan, endothelial cells in red, tumor cells releasing VEGF in orange, and tumor cells not releasing VEGF in blue. In Panel (G), we show the calibrated PDF and the Gaussian fit of the stalk cell growth rate. The mean of the local stalk cell divide time (∼80 hours) is significantly higher than the global stalk cell divide time calibrated at ∼4 hours. This highlights the effect of the hypoxic tumor cells in the top right portion of the domain, as a higher calibrated stalk cell divide time would cause growth toward this region of space, while the data continues toward the bottom right of the domain. (TIF) [file pcbi.1009499.s005.tif]

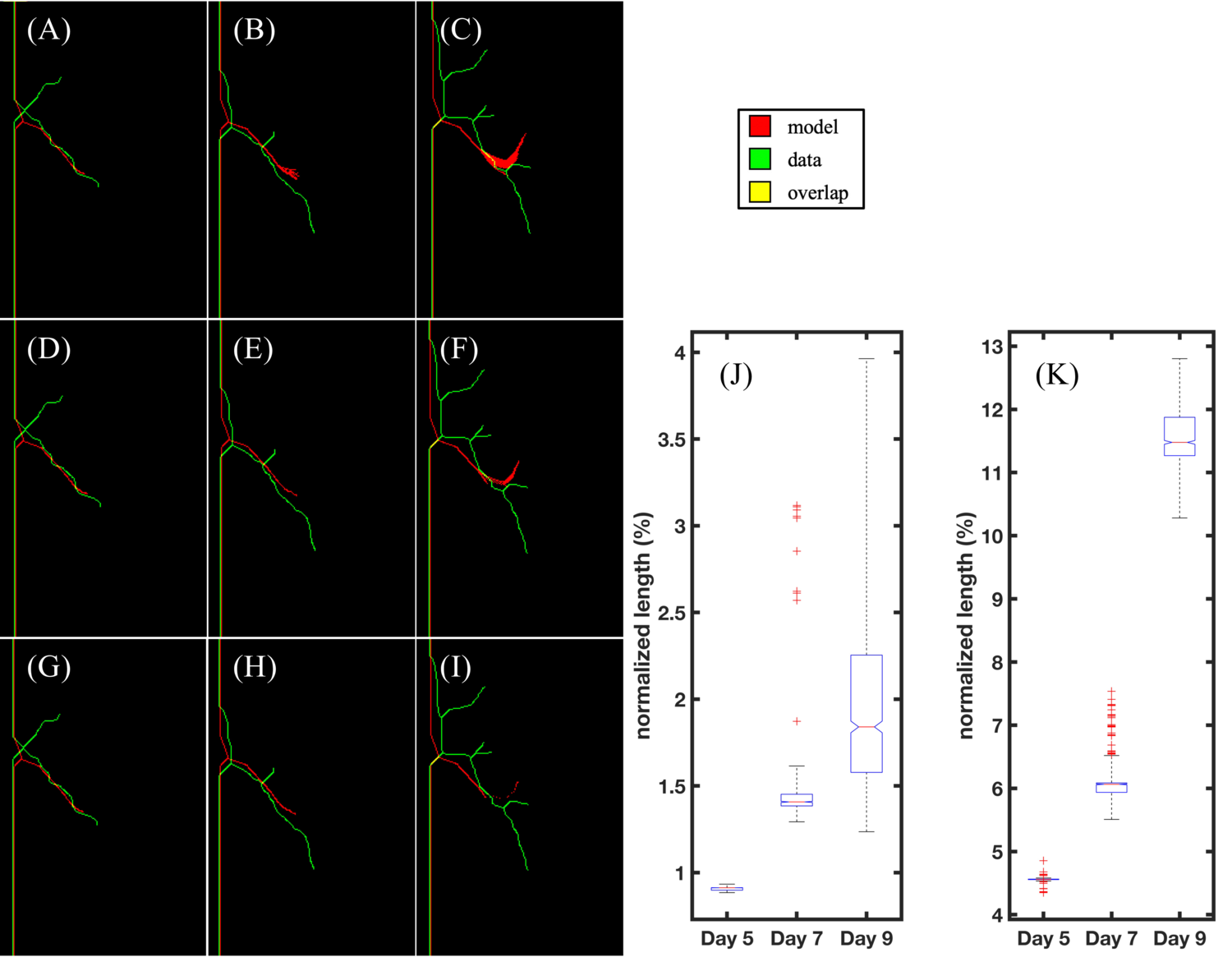

Supplement: S5 Fig — Each column depicts the data (green) and model prediction (red), with overlap in yellow, of days 5, 7, and 9, respectively. Each row shows the voxels predicted by the centerlines using different thresholds, with the voxels in the 99% prediction (top row), 95% prediction (middle row), and 90% prediction (bottom row) of the simulations. In Panel (C), the direction of vessel growth in the model shows the VEGF gradient going away from the direction of data and toward the hypoxic cells shown in Fig 9F. This leads to the overall directionality of the vessel to be misaligned with the data. Panels (J) and (K) shows the prediction of average centerline value from model to data and from data to model with an averaged normalized length difference of less than 2% and 12%, respectively. The average centerline distance is normalized by the length of the longest sprout in this region at day 3. (TIF) [file pcbi.1009499.s006.tif]
