# Supplementary material for: Towards integration of time-resolved confocal microscopy of a 3D in vitro microfluidic platform with a hybrid multiscale model of tumor angiogenesis
Source: PLoS Comput Biol. 2023 Jan 18;19(1):e1009499. doi: 10.1371/journal.pcbi.1009499 (PMC9886306; doi:10.1371/journal.pcbi.1009499)
Supplement: S1 Table — λTp —production rate of VEGF by tumor cells; λec—consumption rate of VEGF by endothelial cells; dSC—stalk cell divide time; dtip—distance between new tip cells. (PDF) [file pcbi.1009499.s007.pdf]

Quantiles	0.05	0.25	0.5	0.75	0.95
λ_T^p	0.0015	0.0016	0.0017	0.0018	0.002
	0.008	0.0012	0.0016	0.0021	0.0024
λ_e^c	0.069	0.082	0.093	0.106	0.127
	0.065	0.080	0.091	0.101	0.117
d_{SC}	17.8	21.0	23.1	24.6	26.5
	18.4	21.0	22.7	24.5	27.0
d_{tip}	224.27	234.15	247.12	254.48	336.59
	222.55	235.18	244.0	252.72	265.3
